# Plasma proteomics-based risk scores for psoriasis prediction: a novel approach to early diagnosis

**DOI:** 10.3389/fimmu.2025.1618805

**Published:** 2025-07-15

**Authors:** Siyu Wei, Zehong Yue, Chen Sun, Yuping Zou, Haiyan Chen, Junxian Tao, Jing Xu, Yuan Xu, Ning Wang, Yan Guo, Qinduo Ren, Chang Wang, Songlin Lu, Ye Ma, Yu Dong, Chen Zhang, Hongmei Sun, Guoping Tang, Fanwu Kong, Wenhua Lv, Zhenwei Shang, Mingming Zhang, Yongshuai Jiang, Hongchao Lyu

**Affiliations:** ^1^ College of Bioinformatics Science and Technology, Harbin Medical University, Harbin, China; ^2^ Department of Medical Engineering, the Fourth Affiliated Hospital of School of Medicine, International School of Medicine, International Institutes of Medicine, Zhejiang University, Yiwu, China; ^3^ Department of Nephrology, The Second Affiliated Hospital, Harbin Medical University, Harbin, China

**Keywords:** psoriasis, plasma proteomics, LASSO, protein risk score model, population attributable fraction

## Abstract

**Introduction:**

Psoriasis is a chronic immune-mediated inflammatory skin disease with a significant global burden. Current risk assessment lacks integration of proteomic data with genetic and clinical factors. This study aimed to develop a plasma proteomics-based risk score (ProtRS) to improve psoriasis prediction.

**Methods:**

Using data from 53,065 UK Biobank (UKB) participants (1,122 psoriasis cases; 51,943 controls), we integrated 2,923 plasma proteins, polygenic risk score (PRS), and seven clinical risk factors. The Least Absolute Shrinkage and Selection Operator (LASSO) algorithm with 10-fold cross-validation identified stable proteins for ProtRS construction. Population Attributable Fractions (PAFs) for risk factors were calculated.

**Results:**

LASSO regression identified 26 highly stable proteins forming ProtRS-26. ProtRS-26 significantly outperformed PRS and clinical risk factors alone. Combining ProtRS-26 with PRS and clinical factors further improved prediction. Key proteins were enriched in pro-inflammatory pathways and skin-derived. PAF analysis identified hypertension and obesity as major modifiable risk factors.

**Discussion:**

Plasma proteomics significantly enhances psoriasis risk prediction compared to genetic and clinical factors alone. ProtRS-26 provides a robust tool for early screening and personalized prevention.

## Introduction

Psoriasis is a chronic, painful, and disabling non-communicable disease mediated by the immune system that manifests as red papules and plaques, usually covered with white or silvery scales (1, 2). Patients may develop chronic inflammatory arthritis and are at increased risk for further cardiovascular and metabolic disease, which can reduce quality of life (3–5). The Global Burden of Disease Study (GBD) database has been established as one of the key research priorities for the global burden by the World Health Organization (WHO) after the publication of data for 2021 showing a global prevalence of up to 42,983,446 (6).

Proteins are direct performers of biological functions and are involved in key processes such as cell signaling, metabolic regulation, and immune responses (7). It has been shown that there is a close correlation between plasma proteins and the risk of a variety of diseases, and protein expression levels can reflect the pathological state of an organism in real time (8). The pathogenesis of psoriasis involves the interaction of genetic, immune and environmental factors, and there is a lack of reliable protein biomarkers to predict disease risk.

**Figure 1:**
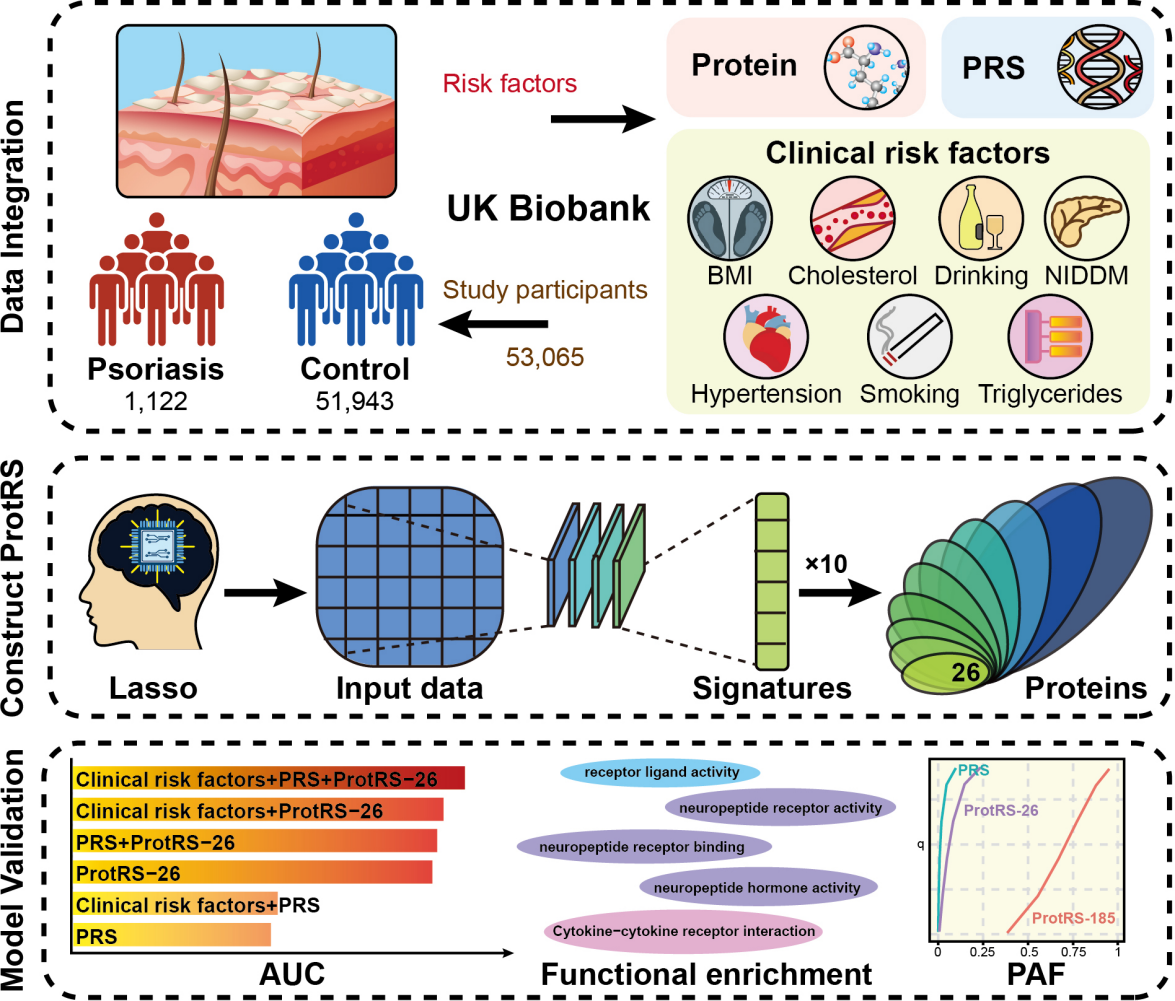
The overall design of the current study. We developed the ProtRS-26 model to effectively predict the risk of psoriasis based on proteomics data from UKB using an advanced Lasso computational strategy. The data included protein expression profiles, PRS and seven clinical risk factors for 53,065 participants. Ten repetitions of Lasso regression analysis were used to screen the characterized proteins to construct the ProtRS-26 model. The predictive effect of the model was finally assessed with AUC, functional enrichment of proteins, and PAF.

Here, we explored the potential of plasma proteomics profiles in predicting the risk of psoriasis (**Figure 1**). We used 2,923 plasma protein measurements, PRS, and 7 clinical risk factors of 53,065 participants from UKB as model features. Modeling the risk of individual outcomes was obtained for 26 proteins by incorporating the proteins into the least absolute shrinkage and selection operator (LASSO) regression model and repeating it 10 times to enhance stability. Finally, the predictive power of ProtRS was compared with clinical risk factors and PRS to broadly explore the proteomic landscape of psoriasis.

## Methods

### UK biobank

UK Biobank (UKB) is a large-scale biomedical database and research platform designed to improve the prevention, diagnosis and treatment of disease. Over 500,000 UK volunteers were collected, with data types including genetics, lifestyle, health records, imaging data and biological samples (9). All participants provided written consent and more detailed information is available at https://biobank.ndph.ox.ac.uk. The present research was approved by the UK Biobank Research and Access Committee, approved application number 89695. The primary outcome was the occurrence of psoriasis, defined based on International Classification of Diseases, Ninth and Tenth Revision, and self-reported disease. The study covered a total of 53,065 participants, including 1,122 with psoriasis and 51,943 as control.

### Proteomics data

Plasma-based proteomics data were obtained from a subset of UKB participants with 2,923 plasma proteins obtained by Olink proteomics assays, data processing and quality control (8). Average interpolation was used to supplement missing values in the proteomics data.

### Clinical risk factors

By summarizing previous studies, we obtained seven clinical risk factors associated with psoriasis, including body mass index (BMI), drinking, hypertension, non-insulin dependent diabetes mellitus (NIDDM), smoking, total cholesterol, and triglycerides (10–16). All of this clinical risk information was obtained from UKB and also covered age and sex. BMI was obtained according to field code 21001; alcohol intake frequency according to field code 20117; tobacco smoking when collecting according to field code 1239; total cholesterol according to field code 23400; and triglycerides according to field code 30870 (**Supplementary Table S1**).

### Polygenic risk score

Polygenic risk score (PRS) is a tool used to quantify an individual’s risk of developing a disease or expressing a trait based on genetic variation. PRS is widely used in the study and prediction of complex diseases by integrating small effects at multiple loci to comprehensively assess individual genetic risk. UKB has published and systematically assessed a standard PRS set of 28 diseases and 8 quantitative traits, with a field code of 26269 for psoriasis.

### Protein risk score

The LASSO regression model was used to reduce the number of features (17). The featured proteins included in the model were finalized by 10-fold cross-validation and selecting the minimum regulation parameter λ for optimal model performance. Protein risk score (ProtRS) was calculated as:


$$ProtRS\;=\;\sum_{\text{i }=\;1}^{n}\;Exp_{i}\;\times \;coef_{i}$$

where Exp is the protein expression, coef is the coefficient, and n is the number of features. To identify proteins with a measure of psoriasis risk, we repeated the LASSO regression 10 times to increase model stability. This was achieved through the R package glmnet.

### Tissue type deconvolution

Deconvolution of tissue types uses the single-sample Gene Set Enrichment Analysis (ssGSEA) method. The featured proteins in the 63 tissue types were obtained from the study by Erik Malmström et al (18). The correlation between protein expression levels and tissue type was used to represent the tissue origin of the protein.

### Assess model performance

In this study, we utilized logistic regression models to assess the performance of ProtRS, PRS, and clinical risk factors, while age and sex were used as covariates for model comparison. To validate the models, 70% of the samples were randomly selected as the train set, and the remaining 30% of the samples were used as the test set. All models were evaluated by measuring discriminative ability through the area under curve (AUC), and efficacy was assessed by odd ratio (OR) and 95% confidence intervals (CI). The DeLong test is used to analyze performance differences between models and is implemented through the R package pROC.

### Population attributable fraction

Population Attributable Fraction (PAF) is used to measure the proportion of a given population in which the burden of disease could be reduced if an exposure factor (e.g., a risk factor, behavioral, or environmental factor) were eliminated. The PAF is an important metric for assessing the potential for public health interventions in epidemiology (19). For binary risk factors, the proportion of the population exposed to the risk factor is calculated to determine the proportion exposed, and the ratio of disease risk for the exposed group to the non-exposed group is calculated to determine the relative risk. For continuous risk factors, the relationship between risk factor levels and disease risk was established to determine the relative risk function, and the overall contribution of risk factors to the burden of disease was calculated by integration. Although the PAF is a hypothetical structure, determining the relevance of specific risk factors to disease and targeting different risk factors for health interventions plays an important role in life health. The R package graphPAF provides support for calculating the PAF for clinical risk factors (20).

### Statistical analysis

All statistical analyses were performed using R software (version 4.4.1) and plots were done with R package ggplot2. Hypothesis tests were 2-sided.

## Results

### Psoriasis study population

This study used 53,065 participants from the available blood proteomics data of UKB, including 1,122 psoriasis patients and 51,943 control participants. We selected seven clinical risk factors strongly associated with psoriasis from a list of selected clinical risk factors, including BMI, drinking, hypertension, NIDDM, smoking, total cholesterol, and triglycerides. Baseline information on the data used was shown in **Supplementary Table S1** and **Supplementary Figure S1**.

### Construction of protein risk score

**Figure 2:**
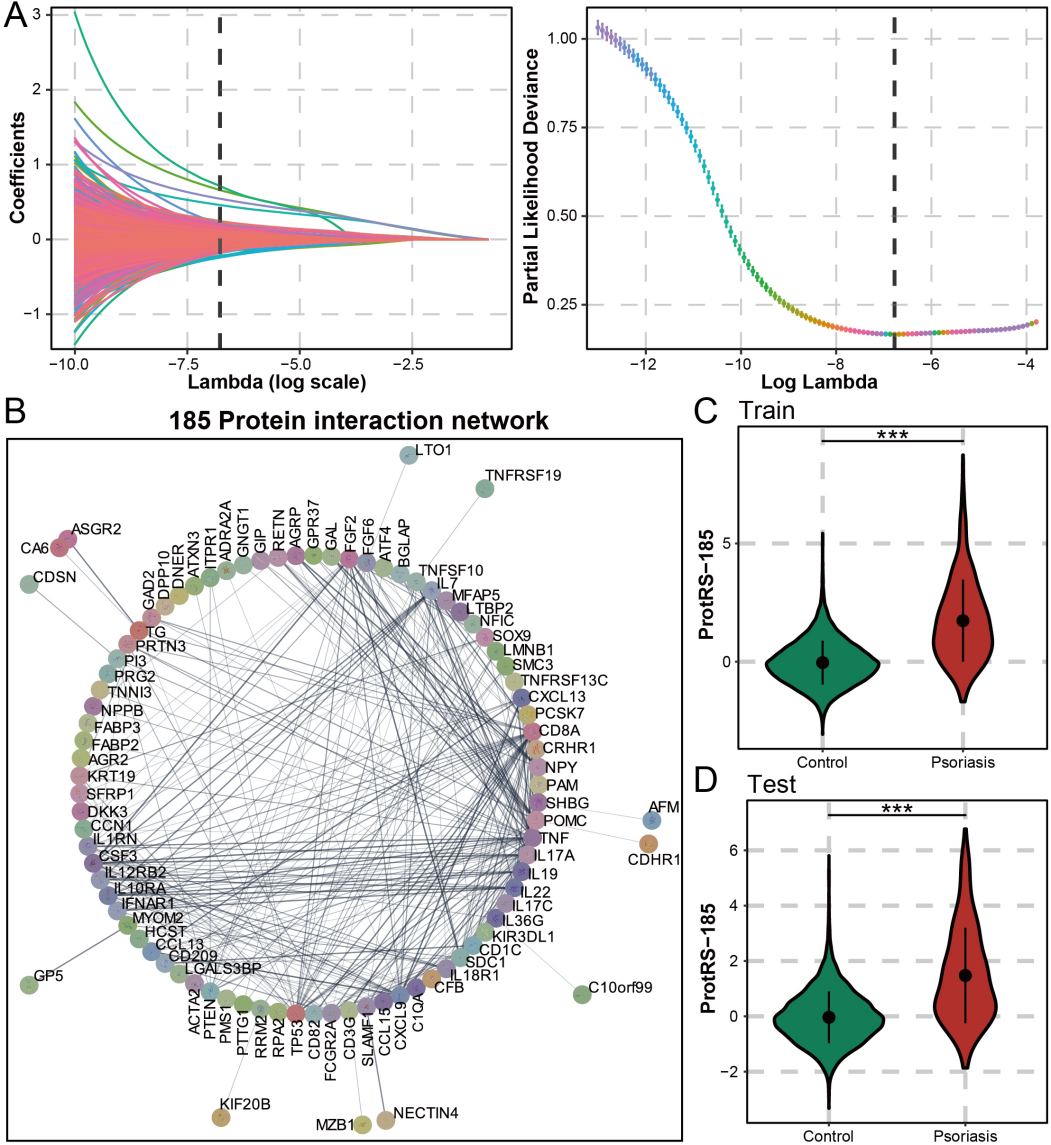
Composition and predictive value of ProtRS-185. **(A)** Construction of Lasso based model to calculate the ProtRS-185. **(B)** Network map showing the interactions between the 185 proteins. **(C, D)** Violin plots showing the differences between ProtRS-185 in psoriasis and control participants in the train and test sets. ***P-value< 0.001.

For the proteomic features of psoriasis, we screened using the machine learning algorithm LASSO regression, which yielded 185 proteins (**Figure 2A**). Protein interaction analysis yielded 14 proteins with more than 15 nodes (TP53, TNF, SDC1, POMC, IL7, IL22, IL17A, IL10RA, FGF2, FCGR2A, CXCL9, CXCL13, CSF3, and CD8A), which may have the potential to serve as a therapeutic target for psoriasis (**Figure 2B**). The clustering results suggested that CD8A, NPY, and TP53 may be interacting (**Supplementary Figures S2A-C**) and were associated with chemokines (**Supplementary Figure S2D**). Furthermore, 60 of these proteins were mainly involved in cytokines related to pro-inflammatory inflammation and interacted with high strength (**Supplementary Figure S3**). Functional enrichment analysis revealed that 185 proteins were significantly associated with the humoral immune response and the regulation of multiple cytokines (**Supplementary Figure S4**). As an immune-mediated inflammatory skin disease, psoriasis was regulated by cytokines and their receptors through a complex signaling network. ProtRS-185 was composed of a weighted sum of 185 proteins. In the training and test sets, ProtRS-185 was significantly different between control and psoriasis, with ProtRS-185 being higher in psoriasis (**Figures 2C, D**). In addition, we constructed predictive models based on clinical risk factors, PRS, and ProtRS-185 using logistic regression and validated them on the test set. ProtRS-185 (OR: 2.922 [2.676, 3.197]; OR: 2.997 [2.661, 3.388]) was statistically significantly associated with psoriasis when the model included ProtRS-185 and age+sex/clinical risk factors (**Supplementary Table S2**).

### Assess the risk of psoriasis

**Figure 3:**
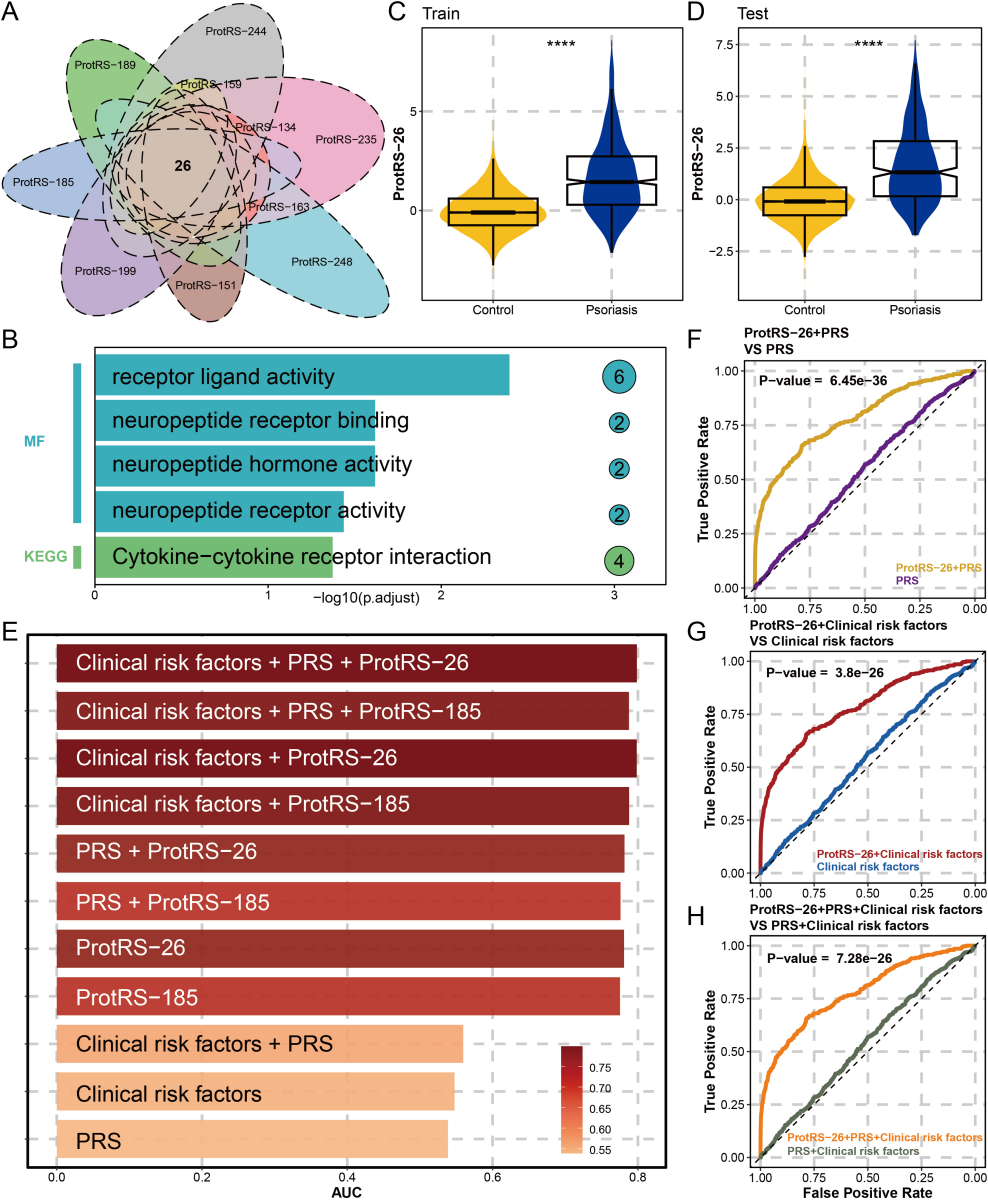
Constructing ProtRS-26 model and analyzing the prediction accuracy. **(A)** Venn diagram showing shared proteins between Lasso regression analyses by 10 resamplings. This intersection led to the identification of 26 proteins strongly associated with psoriasis. **(B)** Functional enrichment plot showing the impact of the 26 proteins in 5 pathways. **(C, D)** Violin plots showing the difference of ProtRS-26 in psoriasis and control participants in the train and test sets. **(E)** Bar plot showing the AUC of the 11 psoriasis risk score models. **(F)** ROC plot comparing the performance between the ProtRS-26+PRS model and the PRS model. **(G)** ROC plot comparing the performance between the ProtRS-26+Clinical risk factor model and the Clinical risk factor model. **(H)** ROC plot comparing the performance between the ProtRS-26+PRS+Clinical risk factor model and the PRS+Clinical risk factor model. ****P-value< 0.0001.

To ensure the robustness of ProtRS, we performed 10 resampling LASSO regressions. The results revealed 104 proteins occurring 5 times and 26 proteins occurring 10 times (**Figure 3A**, **Supplementary Tables S3, S4**). The 26 proteins were significantly enriched for receptor-ligand interactions and neuropeptide-associated functions (**Figure 3B**), as well as being dominated by the pro-inflammatory axis IL36G-IL22-IL19 (**Supplementary Figure S5**), with 20 proteins having significantly different expression levels in psoriatic and control samples (**Supplementary Figure S6**). Based on the results of tissue deconvolution of the psoriasis samples by ssGSEA, 16 of the 26 proteins were probably derived from skin tissues with their expression levels significantly correlated with skin (**Supplementary Figure S7**). Next, we constructed ProtRS with these 26 proteins. In the train and test sets, ProtRS-26 was significantly different between control and psoriasis, with ProtRS-26 being higher in psoriasis (**Figures 3C, D**). In addition, ProtRS-26 was found to differ in age, sex, and 7 clinical risk factors (**Supplementary Figures S8, S9**). We evaluated the predictive ability of ProtRS-26, ProtRS-185, clinical risk factors, and PRS (**Figure 3E**). The risk of developing psoriasis was not accurately assessed using only PRS (AUC: 0.5385) or clinical risk factors and PRS (AUC: 0.5596). In contrast, ProtPS-185 (AUC: 0.7754) and ProtRS-26 (AUC: 0.7809) improved model accuracy by 23% and 24%, respectively. After adding PRS and clinical risk factors as covariates, the AUC could reach 0.7986 (**Supplementary Table S5**). To measure the value of ProtPS-26 as a risk assessment model, we performed DeLong test on the receiver operating characteristic (ROC) curves of ProtPS-26 after adding clinical risk factors and/or PRS. The results revealed that the addition of ProtPS-26 greatly enhanced the prediction accuracy (**Figures 3F-H**). Overall, ProtRS had better prediction performance compared with clinical risk factors and/or PRS, even with the best prediction effect under the combined effect of clinical risk factors, PRS and ProtRS.

### Mechanism of psoriasis-related proteins

**Figure 4:**
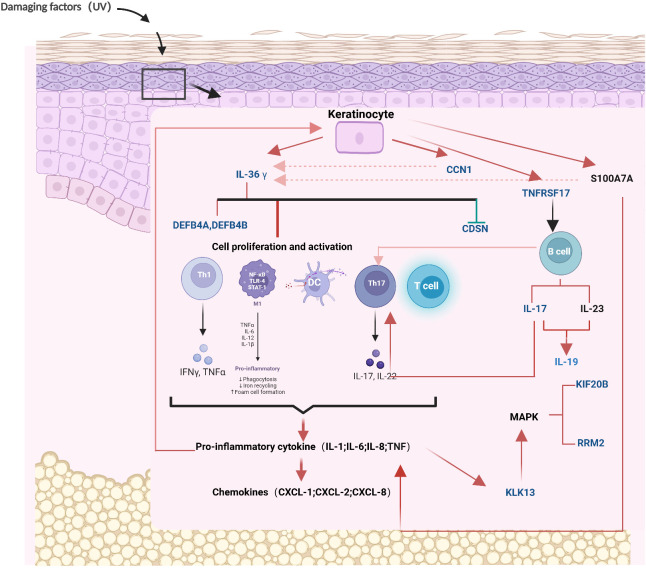
The mechanism diagram of 26 proteins in the pathogenesis of psoriasis. The blue proteins represent the 26 proteins that have been screened out. The red arrows signify promotion, the green arrows signify inhibition, and the red dotted lines denote positive feedback. CCN1, CCN family member 1; CDSN, Corneodesmosin; DEFB4A, Beta-defensin 4A; DEFB4B, Beta-defensin 4B; IL19, Interleukin-19; IL36γ, Interleukin-36 gamma; KIF20B, Kinesin-like protein; KLK13, Kallikrein-13; RRM2, Ribonucleoside-diphosphate reductase subunit M2; TNFRSF17, Tumor necrosis factor receptor superfamily member 17. Mechanism diagram created by BioRender (www.biorender.com), with permission.

Next, the mechanism of the partial protein from ProtPS-26 in psoriasis was described (**Figure 4**). When the skin is subjected to damage such as from ultraviolet rays, keratinocytes express IL-36G (21). Tanel Traks et al. identified the pathogenic role of IL-36, primarily derived from keratinocytes, in the development of psoriasis (22). IL-36G, after cleavage and activation, binds to target cell receptors, stimulates the production of antimicrobial peptides (DEFB4A - DEFB4B) in target cells and down-regulates the expression of CDSN (23–25). Meanwhile, keratinocytes expressed SERPNA1/3, which promoted IL-36G activation. Activated IL-36G prompted the expression of IL-17, which further contributed to the activation of T cells and the production of pro-inflammatory factors (26). These pro-inflammatory factors enhance IL-36G expression on the one hand and inhibit keratinocyte proliferation on the other hand through a positive feedback mechanism (27). In addition, IL-36G upregulates the expression of CCN1 and S100A7A, leading to the production of excessive pro-inflammatory factors by the cells, including IL-1, IL-6, IL-8, IL-36, and TNF, which promotes psoriasis (28, 29). Finally, keratinocytes upregulate the expression of TNFRSF17, which further activates B cells and allows overexpression of KIF20B and RRM2 (30). Notably, RRM2 was identified as the most significant differentially expressed gene between the psoriasis and control groups and is critical in cell proliferation (31).

### Estimate population attributable fraction of psoriasis

**Figure 5:**
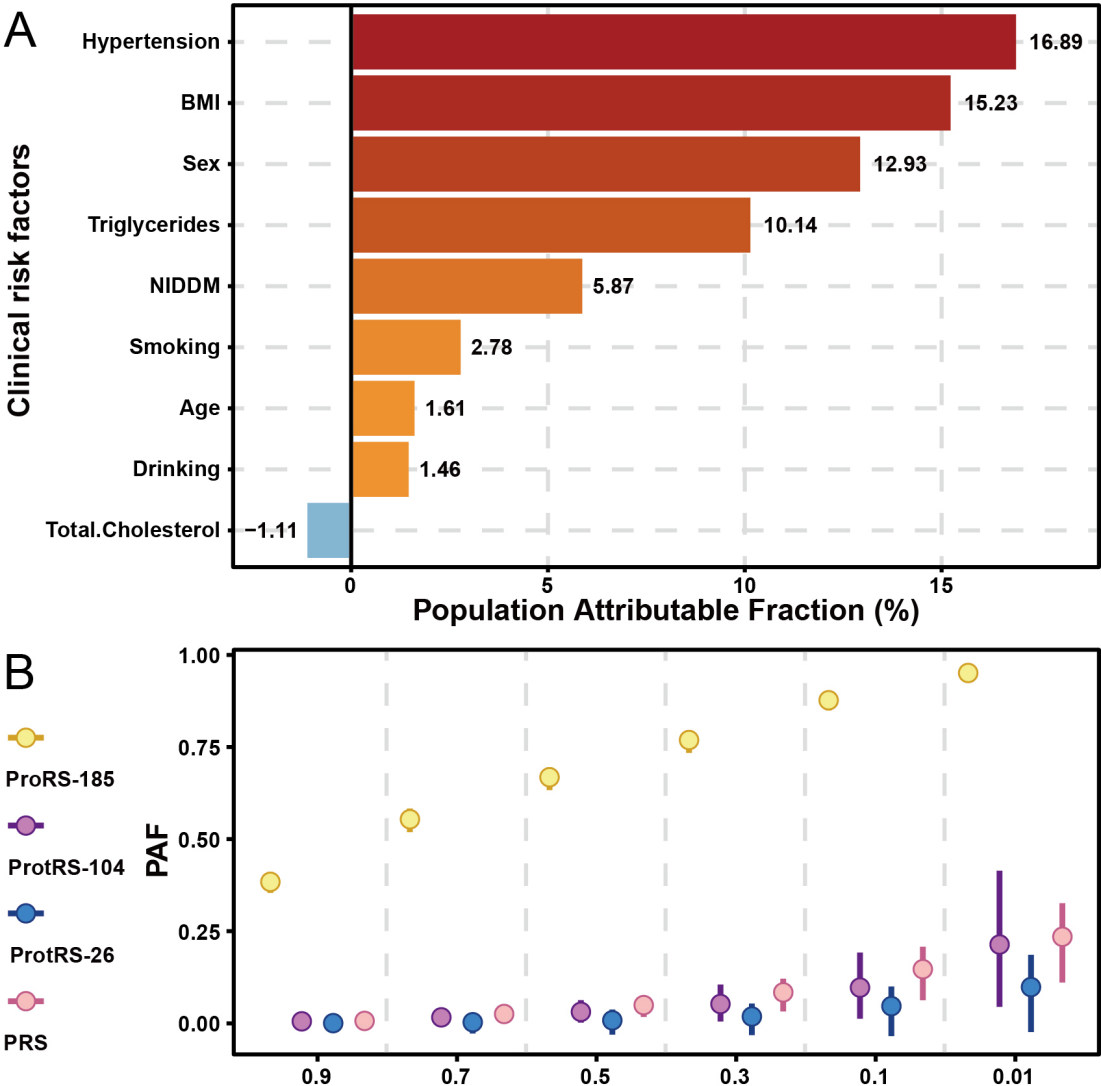
PAF for risk factors of psoriasis. **(A)** PAF of risk factors with age, sex, and clinical risk factors for psoriasis. **(B)** PAF of risk factors with continuous variables for psoriasis.

Hypertension and obesity were the greatest risk factors for psoriasis, with 16.89% and 15.23% of cases attributed to them, respectively (**Figure 5A**). Following this, compared to females, males were at a higher risk of developing psoriasis. The level of triglycerides in the blood also contributed to the development of psoriasis, with 10.14% of the population likely to develop the disease as a result. Some lifestyle habits also increased the risk of psoriasis, such as frequency of drinking and smoking. Intervention at 50% of the most dangerous exposure level may have similar effects on PRS, ProtRS-104, and ProtRS-26, but much greater effects on ProRS-185 (**Figure 5B**). Targeting 185 proteins to return them to normal levels was likely to reduce the risk of disease.

## Discussion

Proteins are central performers of inflammatory and immune responses and have significant advantages as biomarkers in psoriasis research. In this study, we developed a risk score model for psoriasis based on UKB plasma proteomics. The predictive accuracy of psoriasis disease risk can be significantly improved by modeling 26 proteins with LASSO regression compared to clinical risk factors and PRS. Our findings highlight the value of plasma proteomics in enhancing the accuracy of psoriasis risk prediction and provide new insights into the molecular mechanisms underlying this complex immune-mediated disease.

In this study, LASSO regression was selected as the primary model for biomarker selection and risk score construction, guided by the study’s translational objectives and the nature of proteomic data. Plasma proteomics, as a high-dimensional omics dataset, often suffer from multicollinearity and overfitting, which LASSO addresses by applying L1 regularization to shrink non-contributing variables to zero (32). This is a crucial advantage when prioritizing clinically interpretable features over purely predictive accuracy. Furthermore, LASSO’s linear framework provides direct biological interpretability. Each protein’s coefficient quantitatively reflects its weighted contribution to the risk score, aligning with our goal to identify actionable diagnostic candidates. Importantly, the diagnostic performance of our LASSO model suggests that a linear approach to processing of proteomic data provides more significant discriminatory power for psoriasis compared to clinical risk factors.

The 26 proteins identified in our study are enriched for receptor-ligand interactions and neuropeptide-associated functions, reflecting the intricate interplay between immune and neural signaling in psoriasis. Previous studies have demonstrated that the upregulation of RRM2 and CCN1 exacerbates the formation of psoriatic lesions and inflammatory responses by promoting keratinocyte proliferation and the release of inflammatory factors (31, 33). IL-22 has been confirmed to be highly expressed in psoriatic lesions, leading to epidermal hyperplasia and abnormal differentiation (34). A study by Pang-Yen Tseng et al. indicates that GPR15L expression is significantly upregulated in psoriasis and that it has potential as a biomarker of disease activity. GPR15L is mainly secreted by inflammatory keratinocytes, which are highly expressive of differentiation markers and inflammatory factors, suggesting that it plays a key role in the epidermal proliferation and inflammatory microenvironment of psoriasis. In addition, GPR15L can activate sensory neurons thereby inducing itching, and scratching disrupts the skin barrier leading to increased inflammation (35). GAL-1, 2, and 12 of the GAL family have been shown to be potentially associated with metabolic complications of psoriasis, and could potentially be considered as predictors of metabolic disorders leading to renal impairment in psoriasis (36). Increased KIR2DL2 copy number is involved in psoriasis pathogenesis by disrupting immune tolerance and promoting aberrant activation of NK cells or CD8+ T cells. This new finding by Richard Ahn et al. provides a new direction for precise typing and targeted therapy of psoriasis (37). Currently, AGRP, CRIP2, DPP10, FUT3, FUT5, GASK1A, GJA8, GPR37, KIF20B, and MSMB among the 26 plasma proteins have not been found to play a role in psoriasis. This provides a research direction for better understanding the pathological mechanisms of psoriasis at the proteomic level.

Our results demonstrate that ProtRS-26 and ProtRS-185 significantly improved the accuracy of psoriasis risk prediction compared to PRS and clinical risk factors alone. The AUC for ProtRS-26 (0.7809) and ProtRS-185 (0.7754) was substantially higher than that of PRS (0.5385) or clinical risk factors combined with PRS (0.5596). This improvement in predictive performance highlights the added value of incorporating plasma proteomics into risk assessment models. Furthermore, the combination of ProtRS-26 with PRS and clinical risk factors achieved the highest AUC (0.7986), suggesting that integrating genetic, clinical, and proteomic data provides a more comprehensive approach to psoriasis risk prediction.

The PAF analysis identified hypertension and obesity as the most significant modifiable risk factors for psoriasis, accounting for 16.89% and 15.23% of cases, respectively. These findings align with previous studies linking metabolic syndrome and cardiovascular risk factors to psoriasis. The higher risk observed in males and the contribution of elevated triglyceride levels further emphasize the importance of addressing lifestyle factors (e.g., smoking, alcohol consumption) in psoriasis prevention and management. Targeting these risk factors, along with the 185 proteins identified in our study, could significantly reduce the disease burden.

While our study provides valuable insights, several limitations should be acknowledged. Firstly, the UKB cohort primarily consists of individuals of European ancestry, limiting the generalizability of our findings to other populations. Furthermore, while rigorous internal cross-validation was employed to mitigate overfitting, our model has not yet been tested on an independent external cohort from a distinct data source. It is also important to note that, currently accessible large-scale datasets integrating both proteomic profiles and comprehensive clinical risk factors remain scarce beyond the UKB. Future studies should validate our results in more diverse cohorts. Secondly, the cross-sectional design of our analysis precludes causal inferences. Longitudinal studies are needed to establish the temporal relationship between plasma protein levels and psoriasis development. Finally, while our machine learning approach identifies robust protein features, experimental validation is lacking. Their biological relevance and clinical applicability need to be confirmed in independent patient cohorts. Although resource and time constraints precluded these experiments in the current scope, we emphasize that targeted wet-lab validation is a critical next step to translate our findings into actionable insights.

## Conclusion

In this study, the machine learning algorithm LASSO was used to screen proteins significantly associated with psoriasis and to construct ProtRS. The results demonstrated that plasma protein-based ProtRS-26 was effective in predicting the risk of psoriasis disease compared to clinical risk factors and PRS. Notably, the combination of ProtRS-26, PRS and clinical risk factors provided greater improvement in prediction when combined. This has an important guidance in psoriasis prevention, early screening and personalized medicine.

## Data Availability

The original contributions presented in the study are included in the article/**Supplementary Material**. Further inquiries can be directed to the corresponding authors. The data used in this study (including phenotypic and proteomics data at the individual level) were primarily obtained from the UK Biobank, application number 89695, available via the UK Biobank (https://www.ukbiobank.ac.uk/).
