# Supplementary material for: Plasma proteomics-based risk scores for psoriasis prediction: a novel approach to early diagnosis
Source: Front Immunol. 2025 Jul 15;16:1618805. doi: 10.3389/fimmu.2025.1618805 (PMC12303817; doi:10.3389/fimmu.2025.1618805)
Supplement: Supplementary file 2 [file Image1.pdf]

Supplementary Figures

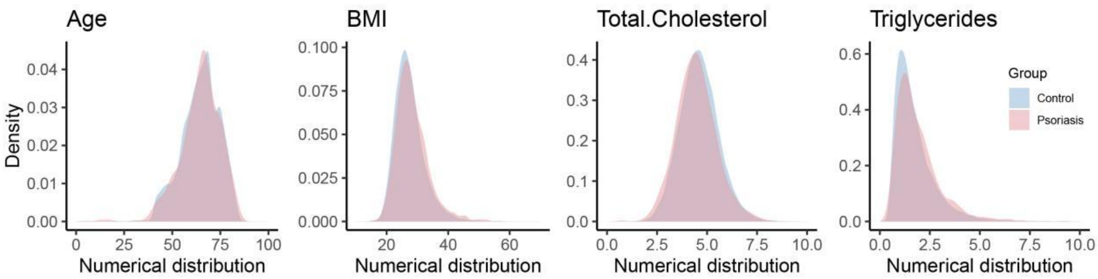

Supplementary Figure 1. Distribution of age, BMI, total cholesterol levels, and triglyceride levels for 53,065 participants in UK Biobank.

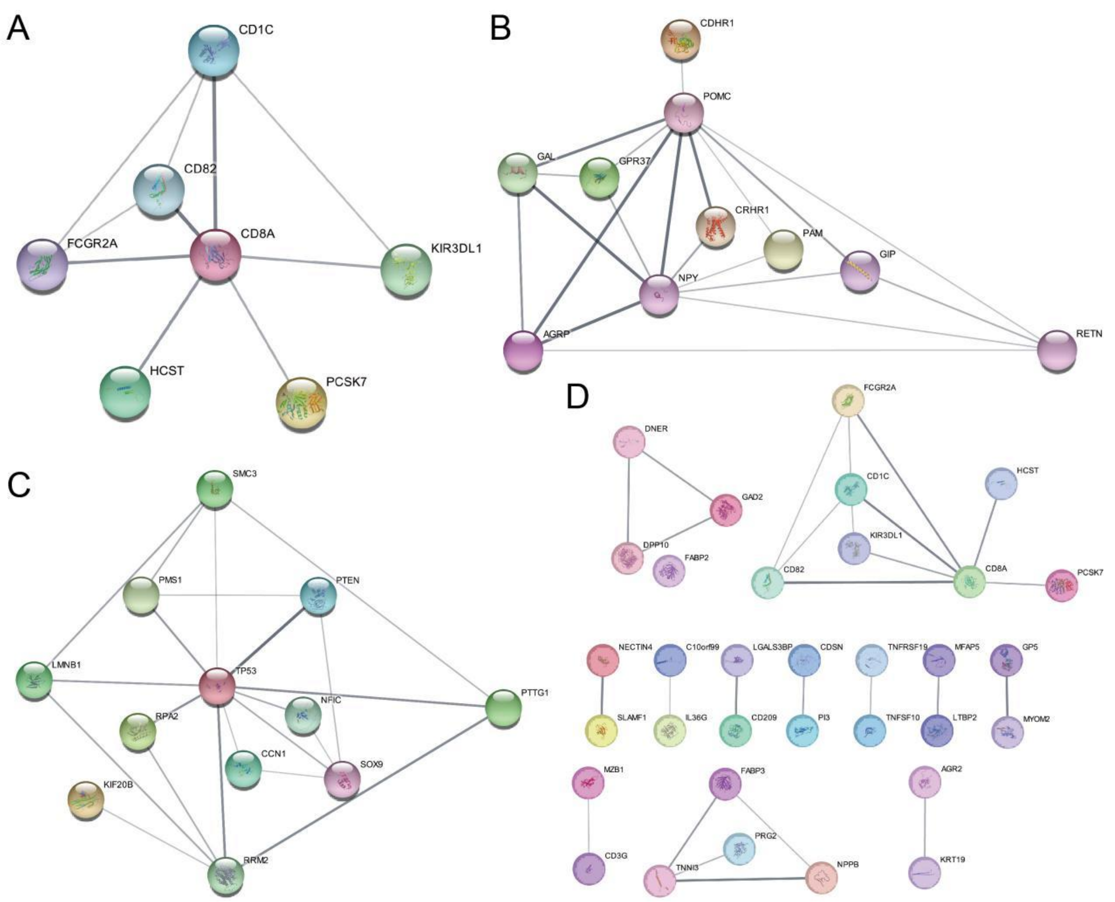

Supplementary Figure 2. The protein interactions were analyzed for 185 proteins with a cluster number of 4. A, B, and C show network maps with nodes CD8A, NPY, and TP53, respectively. D represents the network map of protein interactions of 185 proteins related to chemokines.

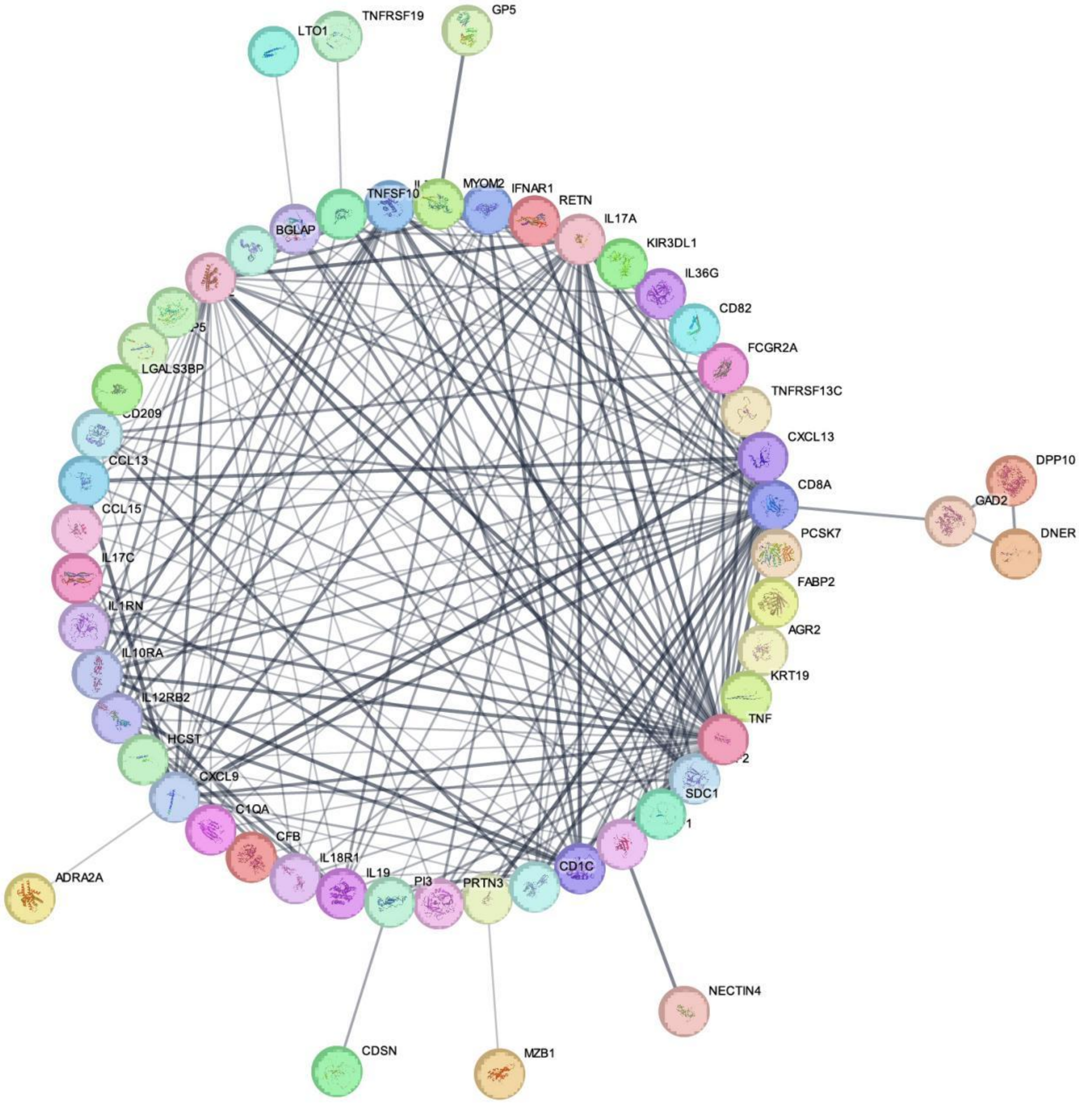

Supplementary Figure 3. The protein interaction network map of 185 proteins subjected to cluster number 3 contains 60 proteins, where the thickness of the line represents the intensity of protein interactions.

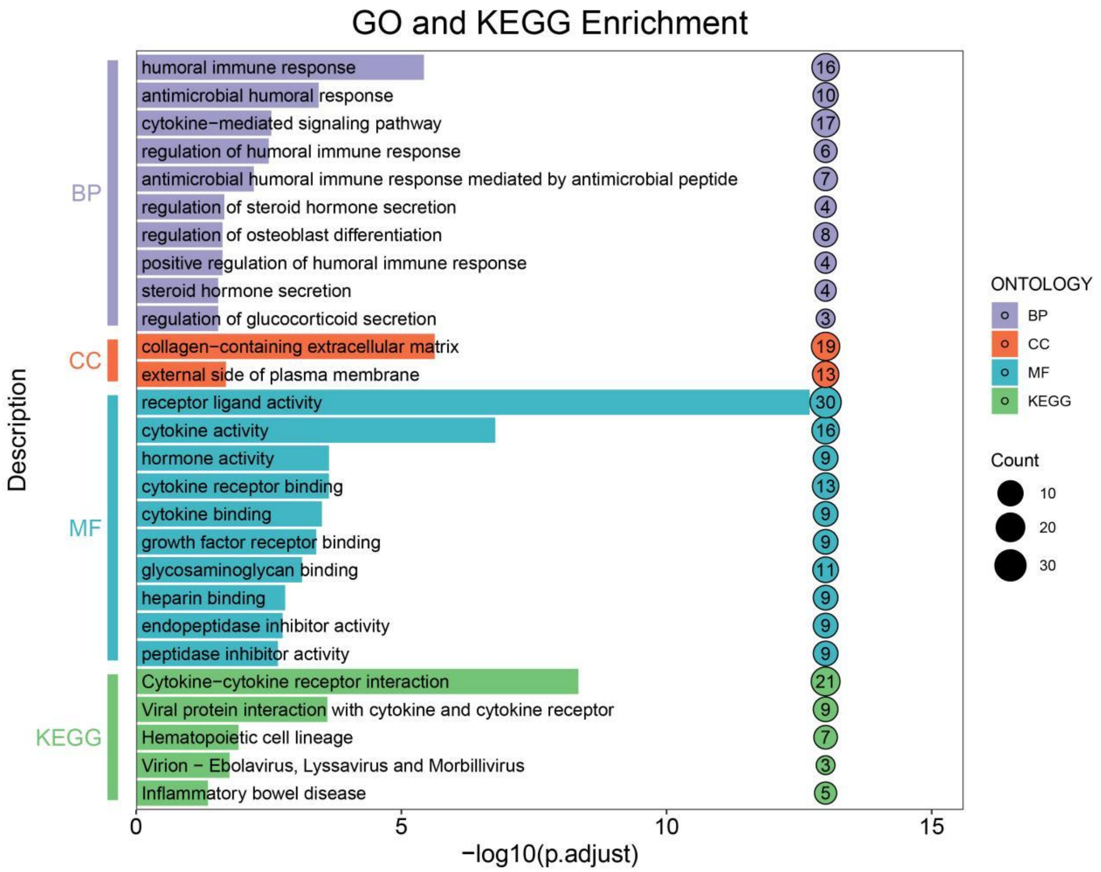

Supplementary Figure 4. Results of GO and KEGG functional enrichment analysis of 185 proteins.

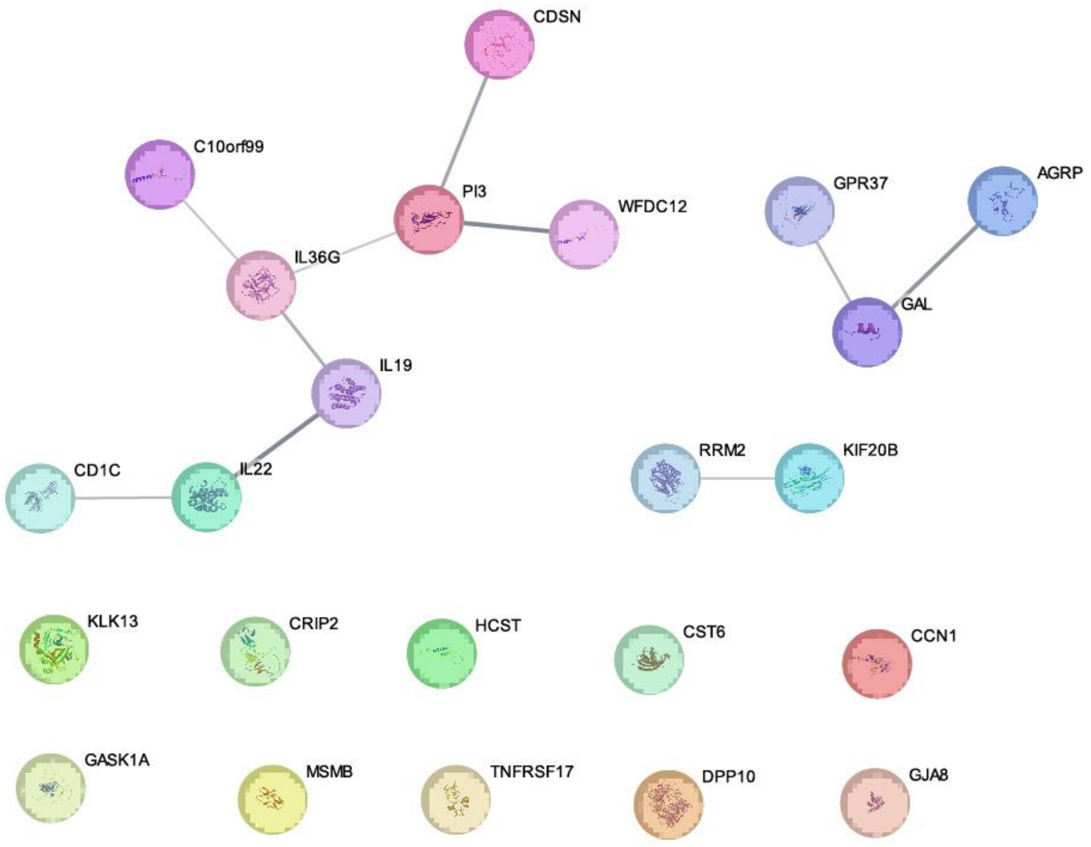

Supplementary Figure 5. Interaction network map of 26 proteins, dominated by the pro-inflammatory axis IL36G-IL22-IL19.

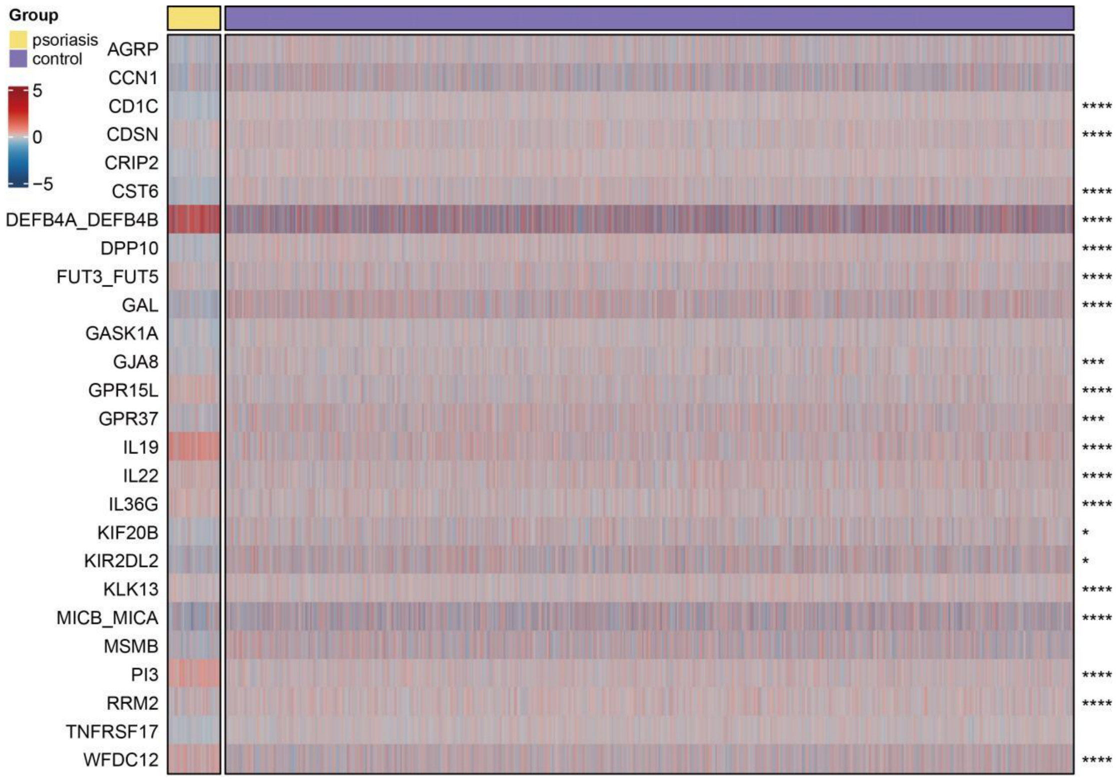

Supplementary Figure 6. Heatmap demonstrating the difference in expression levels of 26 proteins in psoriasis and control samples. *P-value < 0.05, ***P-value < 0.001, ****P-value < 0.0001.

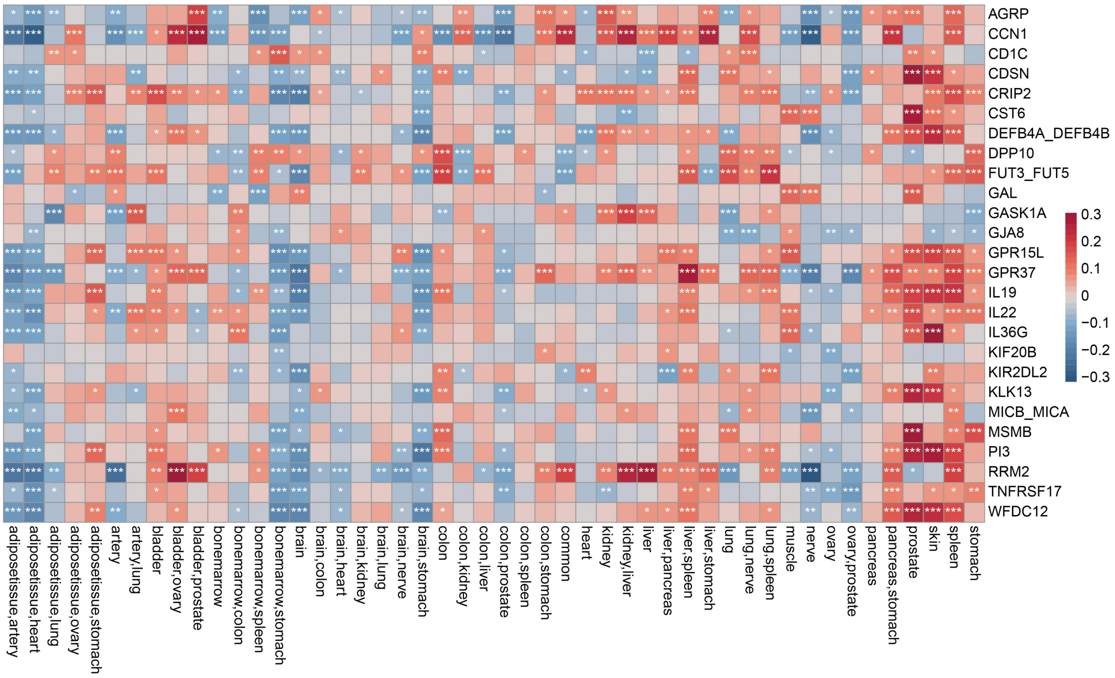

Supplementary Figure 7. Heatmap demonstrating the correlation between the expression levels of 26 proteins and 48 tissues. *P-value < 0.05, **P-value < 0.01, ***P-value < 0.001.

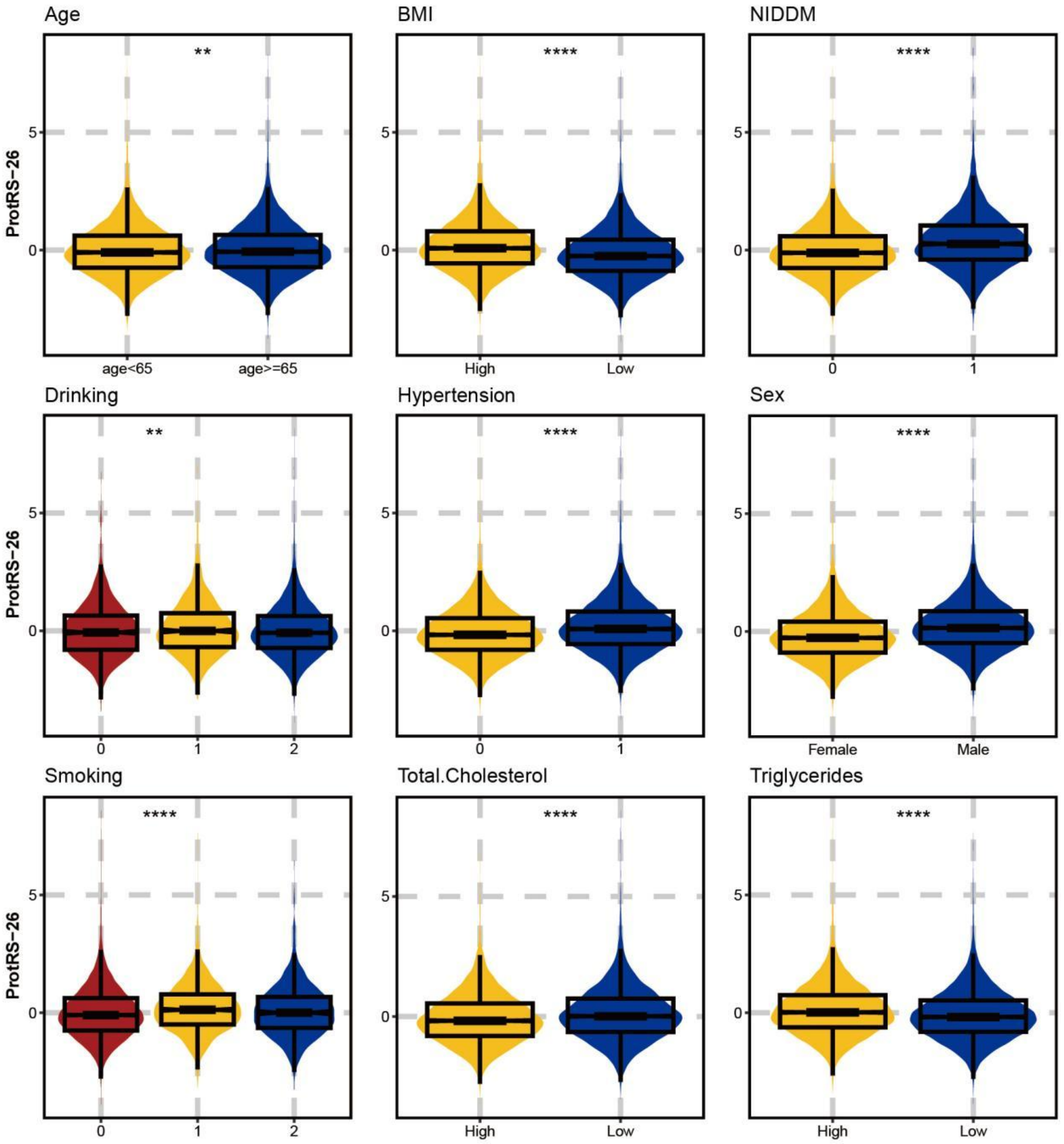

Supplementary Figure 8. Violin plots of ProtRS-26 differences in age, sex, and 7 clinical risk factors in the train set. In NIDDM and hypertension, 0 represents the normal population and 1 represents the disease. In smoking, 0 represents no, 1 represents on most or all day, and 2 represents only occasionally. In drinking, 0 represents never, 1 represents previous, and 2 represents current. **P-value < 0.01, ****P-value < 0.0001.

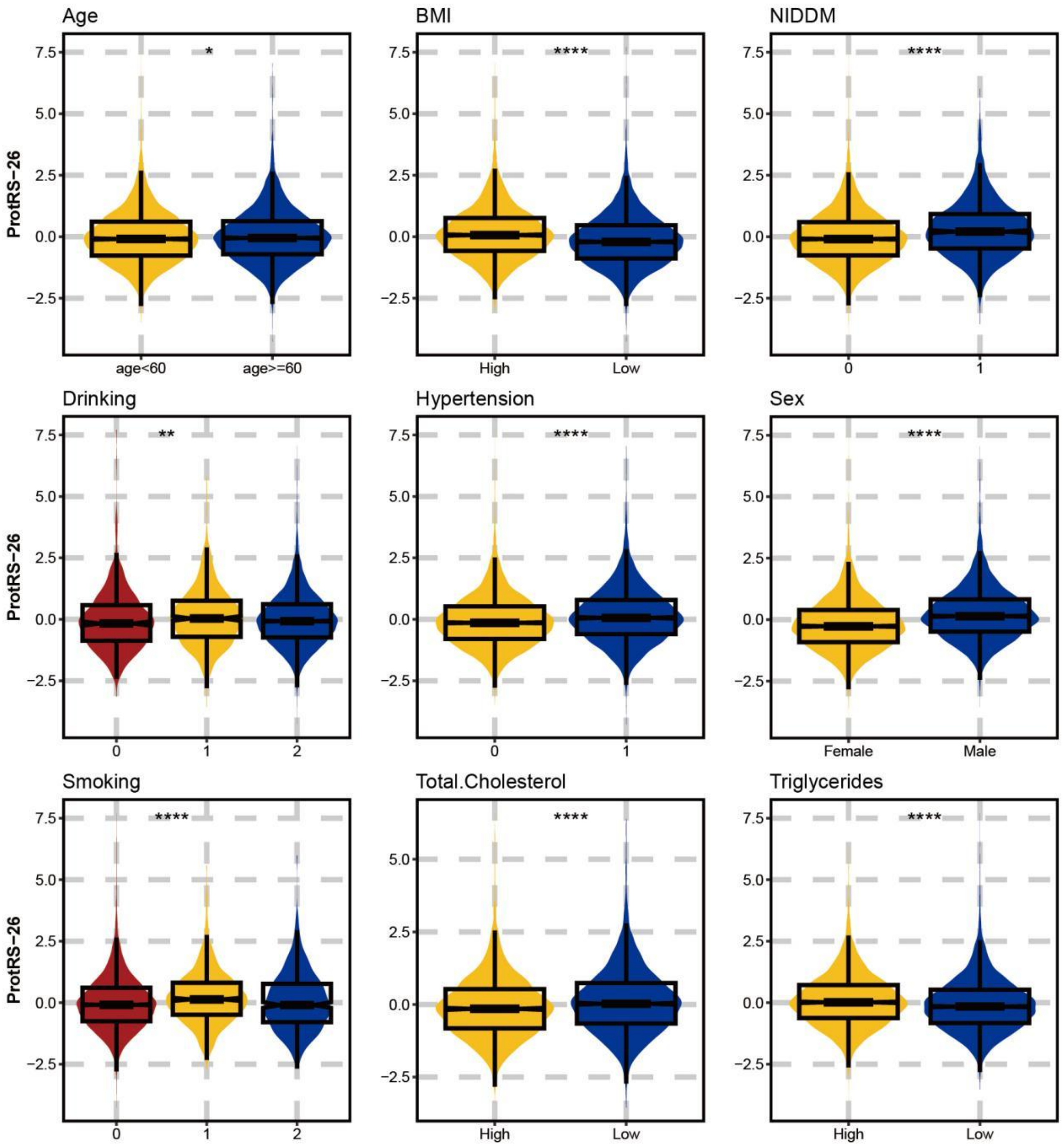

Supplementary Figure 9. Violin plots of ProtRS-26 differences in age, sex, and 7 clinical risk factors in the test set. In NIDDM and hypertension, 0 represents the normal population and 1 represents the disease. In smoking, 0 represents no, 1 represents on most or all day, and 2 represents only occasionally. In drinking, 0 represents never, 1 represents previous, and 2 represents current. *P-value < 0.05, **P-value < 0.01, ****P-value < 0.0001.
